# “They aren’t all like that”: Perceptions of clinical services, as told by self-harm online communities

**DOI:** 10.1177/1359105318788403

**Published:** 2018-07-19

**Authors:** A Jess Williams, Emma Nielsen, Neil S Coulson

**Affiliations:** 1 Nottingham Trent University, UK; 2 University of Nottingham, UK

**Keywords:** clinical services, internet, online communities, self-harm, thematic analysis

## Abstract

Self-harm is a critical public health issue, with strikingly low rates of attendance to clinical services. By offering support, anonymity, and open discussions, online communities hold useful insights into the factors which influence help-seeking behavior. We explore the perceptions of clinical services in three self-harm online communities to understand which services are being used and why. Message threads from each community were extracted randomly until saturation, providing 513 messages across 60 threads. A thematic analysis was performed resulting in four key themes: access to appropriate services during an episode of self-harm, service preference, fears surrounding disclosure, and support.

## Introduction

Self-harm, self-injury, or self-poisoning, irrespective of suicidal intent (National Collaborating Centre for Mental Health, 2011), is known to be a critical public health issue (Hawton et al., 2012). Across ages, it has been estimated that 4.9 percent of the English population will have experienced self-harm, this being slightly higher for women (5.4%) than for men (4.4%) (McManus et al., 2009). Self-reported rates of current self-harm are markedly higher in younger populations, around 10 percent of young people (15–16 years) report engaging in self-harm behavior at some point in their adolescence (Hawton et al., 2012), with rates (of non-suicidal self-harm) as high as 35 percent within university student samples (Gratz, 2001). Repeated self-harm is common (Orlando et al., 2015). As well as being indicative of continuing psychological distress, this is of concern given established risks of escalation as repeated self-harm may lead to increasingly severe medical consequences (Chan et al., 2016; Hawton et al., 2003). The issue is highly stigmatized (Long et al., 2013); consequently, self-harm often remains a hidden behavior (McDougall et al., 2010). Finally, on top of these worrying factors, self-harm behavior has also been established as the strongest predictor of suicide (Bergen et al., 2010; Sakinofsky, 2000); therefore, no incident of self-harm should be taken lightly. For example, those who self-harm are 50 to 100 times more likely to die by suicide in the 12 months following a self-harm episode (National Institute for Health and Care Excellence, 2013).

A major challenge that is faced when addressing self-harm is that the majority of individuals who self-harm rarely present to clinical services, if at all (Arensman et al., 2017; Geulayov et al., 2018), and of those that do re-attendance rates are strikingly low (Michelmore and Hindley, 2012). This low attendance has, broadly speaking, been discussed in the literature within the context of two over-arching themes, the first being the largely negative attitudes self-harming populations feel that health professionals have toward them (Saunders et al., 2012), whereby health professionals are viewed as being dismissive of mental health issues (Biddle et al., 2006) and that these professionals have poor communication abilities regarding self-harm (Taylor et al., 2009). Such experiences have led to reports that health professionals intentionally withhold particular treatments, such as pain-reducing medications during wound treatment, due to presentation being related to self-harm (Owens et al., 2015; Anonymous, 2016). These actions were cited as being related to professionals believing that these patients enjoyed pain (Anonymous, 2016) or that they were less deserving of treatments (Owens et al., 2016).

The second reason for non-attendance relates to the general stigma surrounding self-harm from community populations (Long et al., 2013). Public stigma can lead to self-stigma, which is known to limit help-seeking for general mental health issues (Bathje and Pryor, 2011; Link et al., 2001). This may translate to individuals not seeking self-harm support particularly in clinical services (Crisp et al., 2005; Ross and Goldner, 2009) because they view their injury as not warranting medical attention. Similarly, in obesity, it has been shown that stigma and weight bias cause patients to experience stress around seeking help for a particular condition or medical check, such as a cancer screening (Phelan et al., 2015). This often results in avoidance behavior and late presentation to services (Phelan et al., 2015).

The reasoning and dynamics behind presentation to clinical services are complex (Crisp et al., 2005; Long et al., 2013; Michelmore and Hindley, 2012; Owens et al., 2015; Ross and Goldner, 2009; Saunders et al., 2012; Taylor et al., 2009). While the studies above provide broad insights into this, there are many other factors and barriers which must be identified and understood before we can seek to effectively increase attendance and provide the services that these individuals desire and need.

To gain such an insight, one needs a resource where individuals discuss freely and openly their views on self-harm and health services. The popularization of the Internet over the past few decades has created such a resource in the form of health-based “online communities,” where users with similar health conditions are able to anonymously discuss their views and experiences. Online communities have been used by many authors to explore how people discuss various different conditions, such as irritable bowel syndrome, breast cancer, or Parkinson’s disease (Attard and Coulson, 2012; Coulson, 2005; Wicks et al., 2012), as well as mental health, such as depression (Ybarra et al., 2005). The common themes of communication are health information seeking, discussion of clinical experiences, and support from others who have first-hand experience with the illness (Wright and Bell, 2003). This has highlighted that illnesses or behaviors which might be thought of as embarrassing or stigmatized are more openly discussed. For example, in depression and anxiety online communities, Ybarra et al. (2005) found that conversations between members were more likely to discuss very personal topics and suggested that this was related to the anonymity which is offered by the Internet. Unsurprisingly, these online communities have a strong draw for those who self-harm, in particular as an alternative to clinical services for support seeking (Coulson et al., 2017; Lewis and Michal, 2016). Notably, the asynchronous nature of Internet communication means this support is provided across global time-frames with additional anonymity of their behavior or identity (Suler, 2004).

From the perspective of the researcher, online communities overcome a number of the limitations associated with traditional approaches to data collection. For example, any dataset which involves a large degree of face-to-face interactions will be subject to some level of social desirability (Henderson et al., 2012), especially for a highly stigmatized topic like self-harm. In contrast to the guided technique used in surveys and structured or semi-structured interviews, where the discussion outlines are (to varying degrees) predetermined, online communities allow researchers to view communications as they occur organically between members of the communities and understand how topics and discussions begin, develop, and conclude. More generally, computer-based communication has been shown to encourage people to speak more openly about their emotions and experiences (Rheingold, 1993; Wright, 2000), which is seen in online communities via the spontaneous writing of users and cultures that develop throughout each community. Furthermore, 23 percent of individuals aged 18–29 years stated that they often use online discussion forums, a higher rate than that of any other age group (Duggan, 2015). Therefore, it is highly likely that these online communities are capturing the ages at most risk of self-harm, particularly as we know that young people between 16 and 25 years consistently have the highest figures of Internet usage (Office of National Statistics, 2017), which emphasizes the pool of potential information which can be obtained from these sources.

Across self-harm populations, a few researchers, such as those involved in the forum “Sharptalk” studies (Jones et al., 2011; Owens et al., 2015, 2016; Sharkey et al., 2011, 2012; Smithson et al., 2011a, 2011b), have explored attitudes toward clinical services through engagement with online communities. Owens et al. (2015) utilized a private study forum (“Sharptalk”) to explore barrier breakdowns between young peoples’ and health professionals’ communication when it came to self-harm. While the health professionals recruited were reluctant to engage with the young people within the online community, the young people took the opportunity to discuss their experiences of primary care and how this impacted their self-harm (Owens et al., 2015). In their secondary analysis of this data, Owens et al. (2016) suggest that presentation to emergency services brings with it a “cycle of self-harm, shame and avoidance” (p. 289), which essentially highlights the negative position an individual is already in when they present to hospital with self-harm and how the behavior of the health professionals may further influence this. They discuss how, from the perspective of the doctor, having a private waiting area for a young person who has self-harmed, allows them “privacy,” whereas from the individual’s perspective, they feel they have been set aside, which can potentially allow further self-harm to continue (Owens et al., 2016). This is a difficult relationship to understand, and further exploration of how people who self-harm discuss clinical services may provide greater clarity.

These “SharpTalk” studies sought to gain information from one closed online community designed specifically for their study. This allowed them control of the environment and population, for example, sampling adolescents exclusively and encouraging input from health professionals. In contrast, we seek to gain a more generalized understanding of self-harm and interactions with clinical services and not focus on just one self-selected group of individuals or one community. Therefore, the aim of this study is to explore the perceptions of clinical services within self-harm online communities, in particular: (1) their attitudes toward clinical services, (2) their reasons for choosing not to seek help and, (3) of the subset that do seek help, how their views of clinical services differ and what value they find in these services. By exploring the opinions and views of self-harming individuals, we identify themes that both build on the findings of the studies above, as well as offering a unique perspective of how self-harming individuals view clinical services in an organic environment. This study therefore aims to provide a clear view of some of the most fundamental views and biases self-harming individuals hold toward healthcare, thereby providing a logical starting point for the initial steps one can take to increase attendance and offer a more desirable service.

## Method

### Selecting online communities

This study used a qualitative analysis of messages posted to three self-harm online communities. The inclusion criteria were that the online communities needed to be (1) dedicated to self-harm; (2) publicly available, such that messages could be viewed and downloaded without registration to the site; and (3) currently active, as determined by ensuring that the most recent message to the message board was less than 2 weeks old. These criteria ensured a sample of online communication which was focused around self-harm behavior. The communities selected were the top three consistently occurring sites across three popular search engines (Google, Yahoo, and Bing), using search terms such as *self-injury* or *self-harm* with the phrases *message board, forum*, or *discussion*.

### Ethical consideration

This study was granted ethics permission from the University of Nottingham and was conducted in accordance with the British Psychological Society (2017) guidelines for Internet-mediated research.

All data were within the public domain (Sudweeks and Rafaeli, 1996), thereby removing the necessity to collect informed consent from each member of the site. For example, this type of method has a diverse audience from exploring Facebook and Twitter use during times of crisis (Muralidharan et al., 2011) to understanding online product reviews and clinical outcomes (De Barra, 2017). Importantly, as the study is observational in nature, this also ensured that there was no need to interact with online community members, thus safeguarding against any interference within the community; the group dynamics and communication remained unchanged (Winzelberg, 1997).

Confidentiality of all online community members was ensured by removing their usernames and any response abbreviations from messages, meeting the standard required by the British Psychological Society (2017). Further information such as location, pictures, or demographic listings—which could be considered as potentially revealing identifying information—was removed or replaced if context was necessary to understand the passage. Furthermore, it was crucial for the privacy of community members that all messages used for dissemination were untraceable from their original source (British Psychological Society, 2017). This meant that reverse searching was a necessity for all quotes used in dissemination. In some instances, this meant that shorter quotes were taken from messages or that some potentially relevant messages could not be used due to their traceability.

### Data extraction

Within each of the three online communities, threads of messages were randomly selected for analysis. This was achieved by first organizing posts so that they appeared with the most recent as the first page and going backward temporally, then generating a number between the first and last pages of messages and then assigning each thread from that page a number in chronological order. A second number was then randomly generated and the target thread was extracted in its entirety. This ensured that messages could be analyzed within the full context of the discussion, as well as stand-alone quotes. If upon entering a thread, it was discovered that all messages had been deleted, this was noted and the process started again. This was repeated until saturation was met.

### Data analysis

Deductive-inductive thematic analysis (Braun and Clarke, 2006) was utilized to analyze all messages within their thread context and as stand-alone messages, to identify content related to experiences or perceptions of clinical services. A.J.W. was engaged with the data from initial data extraction. When extraction was complete and the data was fully anonymized, all collected data were shared with E.N. From here, A.J.W. and E.N. developed their own frameworks of themes and subthemes. Notes and discrepancies between the two independent frameworks were discussed, taking forward the strongest subthemes both researchers had individually identified and collating them under a major theme which related to the grouped subthemes. The relevant data were observed and closely analyzed to determine an agreed overall framework. To establish reliability between researchers, 10 percent of coded messages were sent to a third researcher (N.S.C.) who was given theme descriptors but was naive to the original thematic development discussions. Within the overall framework, this reached a high level of agreement of 87.5 percent.

## Findings

In total, 60 threads containing 513 messages from 209 unique online community members were extracted retrospectively throughout June 2016. This dataset represented communication in conversational threads created across a 10-year period, from 2006 until the end of June 2016. Threads contained between 1 and 117 messages, with a median of 5 per individual thread. All messages within this manuscript remain with the original spelling and structure as used within the online communities.

Four core themes were identified; (1) difficult to reach appropriate services in a timely manner, (2) access to therapy; through the medical gateway, (3) confidentiality—fear of disclosure and consequences, and (4) value of support. Table 1 gives the subthemes for each theme (see Appendix 1) and shows each theme’s overall prevalence within the sample, with some messages mapping to one or more themes. The denominator in all descriptive factors was the total number of threads which were included in this analysis. Across 70 percent of the threads, at least one message contained information related to one of the four themes discussed, which related to experiences or perceptions of clinical services.

### Difficult to reach appropriate services in a timely manner

This first theme observes the practicality of service access and how this can be influential on help-seeking behavior within self-harm. Messages conveyed opinions of services not being suitable for them alongside the difficulty of finding the right type of support. What was particularly relevant was that the types of clinical services which were considered to be necessary were not available within the timeframe they were most needed: “the offices are so full up round here … they couldn’t take me until the end of January” (ID: 112, B14). This led to the promotion of private or third-sector organizations over traditional medical care.

Many online community members spent time discussing how they were unsure of what services to access during episodes of their self-harm, particularly if primary care services were not available: “then what else is there?” (ID: 126, B18). In some cases, this was paired with a waiting period, which would help their situation in the long term but during the initial episode was of little use. Alternatively, being able to afford clinical services or having the insurance which could access desirable services was not always forthcoming: “I can’t afford my therapist” (ID: 80, B3); “Medicaid doesn’t cover much” (ID: 191, C3); and “I cant really afford to go private unfortunately” (ID: 1, A1).

Included in these communications were messages which argued that the clinical services offered to them were not always appropriate or suitable: “The doctor didn’t give me stitches, so now I just have a hole in my thigh, that occasionally bleeds” (ID: 191, C13); “I don’t want that kind [psychiatric care] of help!” (ID: 55, A12); “medications making me sick” (ID: 78, B17); and “i just don’t want to end up being put into a long term state hospital” (ID: 63, A15). In response to these messages, other community members often replied by encouraging the member to find an alternative medical professional: “it’s awful that the doctor didn’t give you stitches—can you see another doctor?” (ID: 191, C13) and “you can then choose someone who suits you, you may have to see and few to decide who is right for you” (ID: 2, A1).

Alongside this, promotion of third-sector or private health services was often offered for future reference: “Maybe the Samaritans” (ID: 57, A15) and “Have you tried therapy? It could maybe really be of benefit to you” (ID: 186, C10). In many cases, these were seen as more accessible or as a viable alternative to medical services. Notably, the attitudes toward these services were much more positive, acknowledging differences between particular individuals: “I’m sorry you haven’t had luck with therapists … But really, they’re not all like that” (ID: 83, B6).

### Access to therapy; through the medical gateway

While presenting at medical care was a start, the help that was truly needed was often seen as an understanding of how to cope with underlying psychological issues. Receiving support for this was noted as critically important to reducing self-harm and messages would frequently state that going through a GPs office or medical service (such as the Emergency Department) was the only way to reach this support. Communication circled around how a discussion of the underlying issues which influenced self-harm was the most effective service to reducing their distress “Getting therapeutic help was a deciding factor in my stopping the SH behaviour, and my therapist has been very understanding, accepting and supportive” (ID: 58, A18).

Throughout the messages, therapy was seen as being central to recovery, either as a solution to reduce self-harm itself or as a means to reduce the impact of mental health: “If you approach your gp about you sh or feelings … they may be able to get you some cbt counselling, which you may find helpful” (ID: 2, A1). However, reaching this point often meant the only means of access was via medical services. In this situation, medical services were seen as necessary stages that were required to get to the services which were ultimately desired: “i think you should go back to your GP and explain whats going on … hopefully they can provide you with some more support like a therapy or counselling” (ID: 64, A15).

### Confidentiality—fear of disclosure and consequences

Across this theme, messages discussed not seeking physical healthcare due to self-harm injuries. This was often related to avoiding services because the individuals were concerned about their confidentiality and many users showed a misunderstanding, or uncertainty, as to how healthcare professionals would react to their disclosure. In addition to this, people discussed their fears of being misunderstood at consultations or “labeled” with having a mental health issue, which also appeared to reduce the likelihood of attending clinical services: “I can understand not wanting to be labeled ‘mad’” (ID: 3, A1).

Across messages, there were concerns about confidentiality within clinical services, whether this be attending medical services for self-harm wounds: “If I come in for a broken wrist can they keep me or report me for having cuts?” (ID: 142, C4) or because of assumptions about their mental health: “That said, I don’t want anyone to see it coz they’d probably assume I’m mentally ill or something” (ID: 55, A12). While this subtheme only appeared in 5.0 percent of the threads, the authors felt it was an important finding due to the nature of self-harm and how this behavior is known to escalate. It is important to acknowledge that if these individuals are not seeking help on other matters due to self-injury, then it is unlikely that they will attempt to disclose self-harm at all. In addition, if people are concerned about seeking clinical help because of this fear of self-harm discovery, then there are also implications for accessing healthcare more broadly, such as accessing screenings or seeking medical assistance following accidental injury.

From these, it is clear that the fear of disclosure and the consequences which might follow are a significant concern to this population. This appears to be led by a lack of knowledge about the latter stages of presenting to clinical services and what rights they will have as patients “i’m just scared … I wont be able to get out [of hospital]” (ID: 63, A15). At this point, it is important to note that these online communities are spread globally and that there are thus a range of consequences related to presentation with self-harm across different cultures: “isn’t understood in this country” (ID: 48, A11).

“It’s confidential, as long as you aren’t threatening to kill yourself or others, they won’t tell anyone” (ID: 190, C12), responses to concerns of confidentiality of clinical care staff were ensured by other members. However, despite the large number of positive responses, fear of disclosure and consequences remained throughout the online communities. A small but notable proportion of the messages discussed the fear of sectioning (to be forced into or to remain in hospital due to mental health) or being placed within in-patient services: “second I was found out in my teenage years and subsequently sectioned” (ID: 165, C8) and “they might lock me up” (ID: 1, A1). This spread further than just the initial fear of presenting to clinical services but also to other areas that might be affected by presentation, such as losing their child: “iv been there … and lost my children” (ID: 65, A15), or having to declare self-harm to their employer: “Worried about loosing my job if i have to declare something to my employer” (ID: 1, A1).

### Value of support

As with many online communities, support was commonly sought by those struggling with self-harm from peers who also had similar experiences, and that this was impactful to how they not only handled their emotions: “I think I just need some guidance from people with experience who can help me and be there to talk to” (ID: 76, B2) but also their own injuries. While there was no encouragement of further self-harm in our dataset, some injuries were considered by other members as warranting treatment via self-treatment: “make sure you keep your wounds clean” (ID: 96, B15), whereas others were recommended as requiring professional mental health support or medical services: “Fractures need professionals looking at them, I would not suggest not going because of the cuts” (ID: 143, C4).

Different practices were observed across the online communities in regard to the solicitation and provision of advice regarding the self-management of self-harm. One of the three communities had moderators who deleted messages which contained information about how to look after a wound. In contrast, the other two communities allowed people to discuss how to avoid wound infection: “make sure you keep it clean” (ID: 23, A9) and “dilute the salt alot thow, it will sting ouch but will stop infection” (ID: 31, A9). Self-management, or support seeking within the online community, was also suggested as a means to work through distress and demonstrated that other members understood how the initial member felt: “You’re not alone buddy … I’m here …” (ID: 61, A13). Support was also offered by encouraging members to look how far they’d developed since their last stop-attempt: “… shows that you’ve made some progress!” (ID: 74, B1).

Alternatively, seeking professional help was also frequently seen across messages: “call 999” (ID: 126, B18) and “Be a friend to yourself and see that doctor!” (ID: 97, B15), with members explaining the need for help-seeking in the “real” world; “preferably [counselling] in 3D” (ID: 90, B5). This was particularly relevant when wounds (self-harm or otherwise) were known to be more medically severe: “You can’t leave a broken wrist untreated” (ID: 146, C4).

## Discussion

The aim of this study was to explore the perceptions of clinical services for people who self-harm from the perspective of members of online self-harm e-communities. In order to achieve this, we analyzed user-generated content from three online communities with an international focus. Our findings yielded four key themes, which we will now consider.

Our findings illustrate the challenges of accessing self-harm support services, but capture how peers within online communities can share experiences and advice which may help individuals engage with services. This provides an insight into why some people choose to and choose not to approach medical services following an episode of self-harm.

Specifically, our results demonstrated the importance of finding the appropriate service for an individual and how it needs to be suitable for their personal needs at the time of presentation, whether it is for mental and/or physical support. An evident difficulty which faced this population was that one negative experience, either personal or shared, may discourage attendance at services and lead them to label the service as unsuitable. This can be linked directly to previous findings, which demonstrate the experiences of frustration during hospital attendance (Hunter et al., 2013) due to continual cyclic referrals between GPs and care teams, resulting in large delays or cease actions. The resulting periods of inactivity undoubtedly lead individuals to feel as though a service is unable to handle their behavior or mental state, thereby diminishing their motivation to continue seeking treatment. Avoiding clinical services is therefore, unsurprisingly, considered a viable solution, as the individuals feel the services available to them cannot meet their needs and that attempts to seek help lead to multiple disturbances before receiving treatment. Service barriers within mental health, such as appointment delays, waiting lists, and a lack of resources have previously been shown to be positively associated with avoidance behavior (Kazdin and Wassell, 1999; Spirito et al., 2002).

There is considerable evidence to suggest that stigma and fears concerning treatment negatively impacts help-seeking behavior in young people who self-harm (Fortune et al., 2008; Freedenthal and Stiffman, 2007). Again, it became evident that one negative experience with a doctor, whether it be someone’s own experience or a story from someone else, was a strong barrier to healthcare attendance. This is an issue within other populations such as obesity, where weight bias and perceived stigma from health professionals encourages avoidance behavior (Phelan et al., 2015). Furthermore, these individual cases of “horror stories” appeared to outweigh the influence of the multiple accounts of excellent professional services offered by other members of the online communities. This supports the Owens et al. (2015) “cycle of self-harm” (p. 289), which discusses how young people feel while attending A&E, in particular that they are treated differently. We observed that individuals want to feel that doctors or other health professionals are working with them, rather than for them, supporting the suggestion of Owens et al. (2015) that there could be benefits in evidencing to the individual that they are being treated like any other patient. For example, this could be achieved by giving the patient a more active voice in their received treatments (Michelmore and Hindley, 2012), leading to patient-centered care (Richards et al., 2015). Furthermore, triangulating communications between therapeutic and medical services would also allow for more open relations between people who self-injure and health professionals, hopefully promoting higher rates of help-seeking. Further research would be required to understand whether this solution is a viable, feasible, and acceptable response for those who attend primary care services as well as whether this would be thought to increase future attendance for the population as a whole.

Another important aspect of this study is the understanding of clinical services’ processes and their interplay with client confidentiality. Many users seemed unaware that disclosure of self-harm would, on the whole, remain with only the healthcare provider with whom they had discussed it, or under what circumstances this confidentiality would be broken and how. Indeed, further confusion may well be facilitated by legitimate safety policies, such as discussions within “the team” of health professionals, thus needing clear knowledge of whom this might include. For younger individuals, who are more likely to self-harm (Hawton et al., 2012), this concern is arguably larger, due to the gate keeping guidelines in place for young people and vulnerable populations (National Collaborating Centre for Mental Health, 2004). Young people may worry about their guardians discovering their behavior, how much power they hold over what happens after disclosure and the long-term impact this will have on their social and professional lives. While the responses to these concerns are clear to us, for a young person who is distressed, self-harming and fearful about the services available these are very distinct worries (Michelmore and Hindley, 2012). Therefore, we make a simple recommendation that clinical services state clearly their confidentiality processes in their offices and online (which is more accessible and where a large fraction of this population are spending their time). Here, it is important to stress the differences between an individual attending for help with self-harm and an individual who is in suicidal crisis, stating clearly how this impacts the following steps of care. In line with this recommendation, it would also be beneficial to have a set of standard frequently asked questions about self-harm and how disclosure is handled with that particular clinical service. These will go further in assuring patients of how the interaction will play out without direct input from professionals.

It is also important to discuss the value of group identity, which was evident throughout all of the online communities. When users share a group identity, such as that found on the self-harm sites, the suggestions and recommendations made between users to one another may be more influential. This was illustrated through the observed influence members had on one another when offering advice on self-care and professional help-seeking. Previous research has shown that people often seek support for self-harm through their informal network rather than seeking professional help directly (Biddle et al., 2004; Nada-Raja et al., 2003; O’Donnell et al., 2003), particularly peer support (Biddle et al., 2004; Nada-Raja et al., 2003; Nixon et al., 2008; Ystgaard et al., 2009). Thus, the group support seen in these online communities is both unsurprising and fundamentally important, particularly when it came to influencing a member to approach health professionals for either medical or mental health services.

Qualitative research is a reflective and recursive process (Ely et al., 1991); therefore, we must consider the impact of researcher bias when considering these results. Due to the large pool of previous literature demonstrating that there is a clear divide between help-seeking behavior in self-harm and clinical services, it was acknowledged that this prior information might influence how the authors considered the data during analysis and when reporting. However, multiple authors were required to agree on each theme, including a third author whose primary field is not self-harm. This allowed us to challenge these influences via reliability checking of themes and through discussions of the framework and the final manuscript.

## Limitations

There are a number of limitations of this study which should be taken into consideration. First, our data are taken from three online communities with member representation from many different countries and the organization of healthcare services are likely to vary markedly. Thus, while our findings have captured a range of experiences and viewpoints they are not focused on one single country or healthcare system. Indeed, the nature of our data means that some quotes were limited in their utility within the manuscript due to the identifiable language. However, these quotes were still informative for theme development, and within the manuscript, we adopted similar, less individualized posts to emphasize the meaning and discussed points more generally. Second, our data represent a snapshot or cross-sectional perspective on self-harm support services across a 10-year period. It remains to be seen whether people’s experiences and perceptions changed over time since we did not track the messages posted by individual community members. In addition, while it is considered by some best practice to use “member checking” (Smith and McGannon, 2017) or patient advisory groups to examine the developed themes in a qualitative analysis, this was not done due to the variety of populations across online communities. This was not viable within this context as users of the boards are anonymous and it was important not to join the groups or interact with members as this might have influenced the data. In future, the addition of a PPI group could offer a solution both in terms of checking themes and also thinking about where conversations of this nature are happening (e.g. self-harm specific communities, within schools, or social support). Finally, our dataset may be biased to the extent that those who posted messages about their views on healthcare services may have held a more extreme position than those who did not comment on this issue. Furthermore, from online messages, it is impossible to determine whether a member of these communities is actively engaged with self-harming behavior or if they have dealt with it at all. This limitation is similar to other methods which include self-report responses, although arguably these messages to online communities may have more utility in elaboration than a survey or an interview. This may influence the extent to which these messages can be applied with conviction to a self-harm population at large.

## Conclusion

This study focuses on an issue evident in previous literature, help-seeking, by investigating communication within self-harm populations regarding their experiences with clinical services. The use of online communities provides a unique and organic view of their opinions spanning an international dataset, revealing the difficulties surrounding help-seeking and highlighting why individuals self-select to not attend healthcare. To improve self-harming individuals’ experience at healthcare and increase attendance, we suggest the following: (1) encourage doctors to give patients a more active voice during treatment; (2) make information on how a service will respond to a self-harm presentation (including client confidentiality) easily available and; (3) to consider how the group identity seen in online communities could be used to promote healthcare attendance. Individual responses were also seen to be highly related to the underlying influence of perceived stigma on the part of those who self-injure as well as the external stigma observed within clinical services and the general public.

## Next steps

These results provide a basis for further exploration on how to encourage further attendance to clinical services. In the future, we would seek to assess whether our recommendations could act as a brief intervention to embolden help-seeking. Furthermore, research should focus on understanding how to reduce stigma, both self-perceived and public within self-harm populations.

## Appendix 1

**Table 1.** Thematic framework and prevalence of theme, within self-harm online communities experiences and perceptions of clinical services.

Theme	% of threads containing theme	Subtheme	% of threads containing subtheme
Difficult to reach appropriate services in a timely manner	68.3	Inconsistent or limited access	16.7
		Unsure which services are available beyond GP	10.0
		Unsuitability of services	15.0
		Promotion of third-sector/private care	15.0
Access to therapy; through the medical gateway	31.7	Need for support to stop self-harm (help-seeking)	13.3
		Value of dealing with underlying issue	18.3
Confidentiality—fear of disclosure and consequences	30.0	Not seeking physical healthcare due to disclosure	5.0
		Concerns of confidentiality	10.0
		Fear of labeling/being seen as mentally ill	10.0
		Avoidance responses	10.0
Value of support	41.7	Self-care	10.0
		Support from online peers	35.0
		Encouragement of professional help	18.3

GP: general practitioner.
